# Bibliographical Notices

**Published:** 1854-08

**Authors:** 


### BIBLIOGRAPHICAL NOTICES.

*The Science and Art of Surgery: Being a Treatise on Srugical Injuries, Diseases, and Operations.* By JOHN ERICHSEN, Professor of Surgery in University College, and Surgeon to University College Hospital, London. Edited by JOHN H. BRINTON, M. D. *Illustrated by three hundred and eleven engravings on wood.* Philadelphia: Blanchard & Lea, 1854. Large 8vo. pp. 908.

It is very gratifying to see the growing importance attached to combining instruction in the science as well as the art of medicine. It is in this mode only that true advancement can be gained; it is thus alone that our profession can hope to free itself from the too often just reproach of being guided by routineism and empiricism, rather than by sound and enlightened reason.

Mr. Erichsen is presented to us under very favorable auspices. He has been long and well known by his numerous valuable contributions to surgical literature, and by his position in one of the most promising of the London Schools and Hospitals. He cannot be accused of "rushing unapparelled into print," but is thoroughly clothed and in his right mind.

The volume before us is far too large and comprehensive for anything like an elaborately critical review. We shall, therefore, take the liberty of dealing with it as we should with any other collection of valuables exposed for sale; we shall select a few of what seem to us to be the most desirable and striking objects presented to us, and perhaps chaffer a little as to their worth.

No exposition of the state of surgery would be tolerable which did not start with the consideration of *Inflammation.* For, as Mr. Erichsen truly says, "an acquaintance with its nature, symptoms and progress, gives an insight into a great part of the science of surgery. The management of inflammation as it affects different tissues and organs, and thus constitutes distinct diseases, comprises a great part of the duties of a Surgeon."

Mr. Erichsen precedes the description of inflammation proper by a sketch of two other forms of "*increased vascular action,*" which he terms *congestion* and *determination.* In dealing with the phenomena of inflammation, he describes the state of the vessels, the changes which the blood itself undergoes, and then the symptoms, as they are called, including the various physical alterations of tissue, the modifications of function and of nutrition, and the constitutional disturbances in the numerous grades and types of the disorder; he then enumerates and explains the *termination* and *effects* of *inflammation*, its *causes* and its *treatment*. Next he describes what he regards as the "*Secondary forms* of *inflammation*," the effusive, the adhesive, the suppurative, the ulcerative, and the gangrenous.

This, it will be observed, is the routine method of treating the subject; and although Mr. Erichsen gives the most approved modern views and explanations of the phenomena of inflammation, we do not see that our knowledge of the process is in the least advanced. Some may indeed find fault with the brevity with which the author treats of it; but we are disposed to allow him to decide for himself what is most consistent with the general plan which he has traced out in his own mind. In fact, he signifies his own idea upon this point; he says: "the theory of inflammation is a purely physiological and pathological study; and, however interesting its investigations may be, yet as the discussion of this subject belongs rather to the domain of general pathology than to that of practical surgery, it cannot consistently be entered upon here. But regarding the subject from a surgical, rather than from a physiological or pathological point of view, we must discard all hypothesis, and confine ourselves to the results of trustworthy observation."

An objection which strikes us as being more valid against Mr. Erichsen's chapter on inflammation, is that he does not leave us with any definite impression either as to what is really the nature of the inflammatory process, or as to his own views concerning it. After describing briefly but clearly the chief alterations which inflammation induces in the vessels and the blood, he says, "but it must not be supposed that the process consists simply in these changes; the nerves and parenchyma doubtless exercise an important influence, the precise nature and extent of which can scarcely at present be appreciated, though the observations of Paget and W. Jones demonstrate its existence." p. 140.

Now, if any progress is to be made in our knowledge of inflammation, this certainly is the direction which it is most desirable that it should take. Let those whose position and experience are such as to draw attention to themselves as teachers of medicine, interpret and expound the facts which have already been collected in sufficient abundance. Let them inform us in what respects inflammation differs from healthy nutrition, and then we shall be enabled to combat it with more satisfaction and success: Mr. Erichsen merely repeats in brief a few of the facts which Mr. Paget and Wharton Jones have before told us *in extenso.*

We are convinced that the proper way to study inflammation is to pursue the path which Mr. Paget has opened,—to fix in the mind the elements concerned in the normal process of nutrition, the circumstances and conditions which are most conducive to it, and those which interfere with it; and then to observe how gradually increasing disturbance and modification of the normal conditions lead us to the complex abnormality, *inflammation.* In this way the study is materially simplified; we have already become acquainted with all the factors which are engaged—first in their ordinary avocations, having a strictly normal material to act upon, and their naturally appointed implements and forces to work with, and then with these materials and endowments modified. We thus acquire juster notions concerning the part which each component element of the tissues,—the blood-vessels, the nerves, the tissue-cells,—plays in the processes of nutrition, both natural and morbid, and we shall be less likely to ascribe to any one an undue importance.

It is evident, we think, that erroneous views concerning the nature of inflammation have originated in inattention to the phenomena of ordinary nutrition. Thus the Edinburgh School of Medicine has long contended "that inflammation is the exudation of the liquor sanguinis," (see the Edinburgh Monthly Journal, Jan. 1854; Dr. Bennett's "Treatise on Inflammation as a process of anormal nutrition;" also Miller's "Principles of Surgery.") But the exudation of blood-plasma is essential to the ordinary nutrition of every tissue, the amount being regulated by the requirements of the latter, and varying, therefore, as these vary. Normally, the tissue-cells dispose of the whole of the exuded plasma converting it into cells and fibres, and if the supply be at any time increased, it is so in obedience to an increased demand on the part of the same formative centres, so that nutrition is exaggerated into hypertrophy. Here, then, is an increased exudation, yet no inflammation. Again, we have seen an increased fibrinous exudation in cases of dropsy of the abdomen from diseases of the liver, when neither during life nor after death had we any reason to suspect the existence of inflammation: coagula of lymph were found in the more abundant serous fluid. To constitute inflammation, therefore, we must have not only an augmented exudation of liquor sanguinis, and an altered liquor sanguinis, but also a defect and a modification in the formative power and action of the tissue itself, as is abundantly evident in the degenerative changes produced. And this last element in the process we conceive to be as essential as the other; indeed, we think it should have the preference, as a characteristic of the inflammatory process, inasmuch as in ordinary nutrition, it is the tissue which seems to determine the amount of the exudation.

Another erroneous view, as it appears to us, and one which flows more or less naturally from the opinion which we have just been criticising, is that inflammation is impossible in non-vascular tissues. It is urged by the supporters of this opinion, amongst whom Dr. Bennett is very prominent, where there are no blood-vessels there can be no exudation, and where there is no exudation there can be no inflammation.

But vascularity, like exudation, is merely a relative term. All vascular tissues are not equally abundantly supplied with blood-vessels; nor yet is the same tissue, vascular though it be, under all circumstances permeated by blood-vessels; and, conversely, some tissues which are usually non-vascular, have, under certain conditions, their own system of blood channels. For example, the *osseous* is a vascular tissue, having an especial and a very curious arrangement by which all parts of its structure are rendered accessible to the blood streams; and yet the scale-like turbinate bones have no blood-vessels of their own, but are nourished from those which ramify so abundantly in the mucous membrane covering them; consequently these vessels, though not actually an anatomical part of these bones, yet really belong to them as much as if they did enter into their structure. On the other hand, the *cartilaginous* is not counted amongst the vascular tissues; and yet, when the cartilage is of unusual thickness, as in the case of the costo-sternal cartilages, it is actually penetrated by blood-vessels, in order that its central particles may gain a sufficiency of nutritive material. (Goodsir, Anatom. and Pathol. Observations, p. 17 ; Sharpey and Quain's Anatomy, vol. i. p. 242.) The fact is simply this ; a certain supply of plastic material is necessary to the maintenance and growth of every living organic structure, and the amount of this supply is determined by the requirements of each, the general rule being that the more active the function of a tissue the more abundant its supply of organizable matter, because the greater and the more rapid is the waste of its particles. It is not necessary for the purposes of nutrition that each particle shall be in actual contact with a blood-vessel; the fibrils of the muscles and nerves are richly nourished, yet they are quite remote from the sanguiferous channels. All that is required is that the blood shall be so distributed that the tissue particles may gain by imbibition the materials for their adequate growth and renewal.

Those tissues which are usually called non-vascular do not need to be actually permeated by blood-vessels, because their function is of the most simple and passive character, their particles, consequently, slowly become effete, and are therefore tardily and infrequently renewed. Such are the cornea, the crystalline lens, the vitreous body, and the articular cartilages. But these structures are liable to modifications of nutrition, and hence to quantitative alterations of plastic exudations, just as vascular tissues are, although in a less degree, and therefore their particles are placed merely a little more remote from the blood-vessels, whence they derive their nutriment, than are those of the vascular tissues.

Admitting then that, in the ordinary nutrition of vascular tissues, imbibition of liquor sanguinis takes place sufficient for the requirements of their function and structure, how can it be *impossible* that an increased exudation, or an augmented imbibition, shall occur under the influence of the modified relations which inflammation induces between the tissues and the blood-vessels ?

But, theory aside, we believe that facts prove conclusively that inflammation is not only possible, but of actual occurrence in non-vascular tissues.

The cornea is admitted to belong to this class. It has no blood-vessels of its own, but derives the materials for its sustenance from those of the sclerotica and conjunctiva, which, on arriving at the border of the cornea, turn back, forming numerous arches that run parallel with the corneal margin for some little space, and then return whence they started. We select the cornea for the purposes of our argument, because it is perfectly transparent when healthy, and is accessible to constant observation.

Now, *inflammation of the cornea* finds a place in all our nosological tables, and is considered by most persons to be as much of a pathological entity as inflammation of the brain or liver. Yet Dr. Bennett and his friends would have us to believe that there is no such thing as *corneitis*, properly speaking. Let us see, therefore, if the changes attendant upon certain injuries of the cornea are not identical with those which occur after similar injuries inflicted upon vascular tissues.

If the cornea be punctured or slightly incised, accidentally or by the surgeon, the sequelæ will ordinarily be the following: the bloodvessels of the conjunctiva and sclerotica nearest the wound will speedily dilate and convey a larger quantity of blood and of organizable material; this material will find its way by imbibition to the margins of the wound, which will thereby acquire a nebulous or milky appearance, and if it be examined microscopically it will be found to consist of nucleated blastema, similar to that which, under favorable circumstances, serves as the reparative material of wounds of vascular parts; gradually, and speedily in inverse proportion to the abundance of the deposit, the opacity clears up, the new material acquires the physical properties of the normal corneal structure, the natural transparency of the part is regained, the dilated vessels of the adjacent tissues shrink back to their proper dimensions, and frequently no vestige of the injury remains.

If a more considerable violence be inflicted, the margins of the aperture acquire a greyish and ulcerous look, the opacity from plastic infiltration is much greater than in the first instance, the neighboring blood-vessels become still more enlarged, and the aperture closes slowly by a process resembling that of granulation in vascular parts. Mr. Bowman describes a case in point, in which a piece of caustic potassa had produced a small ulcer on the cornea of a cat. The conjunctival epithelium and the anterior elastic lamina of the cornea had been removed, and the superficial lamellæ of the cornea proper formed the bed of the ulcer. These were softened and semi-opaque from the presence of great numbers of nuclei which were most abundant on the surface of the ulcer, occupying chiefly the position of the corneal tubes—a system of canals peculiar to the cornea, their office seeming to be to convey to the recesses of the structure the plastic material which is derived from the blood-vessels of the adjacent tissues, (Bowman on the Eye, p. 30.) Now, here certainly was a plastic exudation in the substance of a non-vascular structure.

We have seen that under ordinary circumstances the nutrition of the cornea does not require the presence in it of blood-vessels, which would of course interfere with its especial function. But when these normal conditions are changed, and the cornea requires to have its vitality increased and the rapidity of its nutritive actions augmented, then we find that blood-vessels actually become developed in it to meet these new requirements. Thus, if an ulcer has existed for some time upon the cornea, this tunic becomes opaque by the deposition of plastic matter between the ulcer and the dilated vessels of the conjunctiva and sclerotica; and in this organizable matter, new vessels gradually appear sprouting from those just spoken of, and approaching nearer and nearer to the ulcer until they reach this point; now a dense opaque exudation from them fills up the excavation, and the breach becomes healed. Or, when the cornea is constantly fretted by a granular lid, under what other name than inflammation shall we describe the phenomena which ensue—the infiltration of the tissue with organizable material, and the development in it of new vessels, to enable the tissue, whose vitality and power of enduring irritation are small, to sustain and recover from this morbid stimulation? Mr. Bowman has "a specimen in which these adventitious vessels are displayed injected with artificial color; they pass into the cornea from the conjunctiva and the whole thickness of the sclerotica, and occupy, in this particular instance, almost the whole thickness of the lamellated tissue." (Op. cit. p. 31.)

Thus we see, that in the cornea, morbid stimulation is followed by the same effects as in vascular structures. Another proof that the material exuded into its substances is what is commonly called *lymph*, or *plastic matter*, similar to that which is produced in inflammation of vascular tissues, is, that it is subject to the same sort of transformations—the cretaceous, the fatty, and the purulent.

We trust that our readers will pardon this long digression; but the questions which we have raised are of sufficient interest to repay consideration, and we cannot but think that the opinion of Dr. Bennett is wrong upon both of them. We cannot believe that the turbidity of which we have spoken is due to the presence of fatty molecules, as Virchow and others seem to think, or to disintegration of the proper structure of the cornea, in view of the observations of so careful and excellent a pathologist as Mr. Bowman, and of the reparative and organizing processes which we have traced in them, which are inexplicable upon the supposition that there has been no plastic deposit.

After treating of inflammation and its sequelæ, Mr. Erichsen devotes several pages to certain "general considerations on operations," comprising a statement of the conditions which are favorable and unfavorable to their success; the causes of death; the circumstances to be attended to preparatory to their performance; the use of anæsthetics; the after-treatment of operations, &c, &c. Next, between twenty and thirty pages are allotted to the subject of "*Amputations* and *Disarticulations*." We need only say that the author exhibits all the practical skill and good judgment which we had expected to find displayed in his discussion of these important topics.

"*Surgical Injuries*" are fully described in the second division of the book, extending over two hundred of these large and closely printed pages.

In the section on "*Wounds*," Mr. Erichsen enumerates five modes in which wounds heal, viz: "by the direct growing together of their opposed surfaces; by scabbing; by the opposite surfaces uniting through the medium of coagulable lymph; by granulations springing up from the sides and bottom, and thus filling up the wound; and by growing together of two granulating surfaces." The third method, the union of the opposite surfaces by the intervention of lymph, he also terms "*union by the adhesive inflammation*;" and after clearly stating the part which this plastic exudation plays, in the process of repair, he says, "for the production and organization of this lymph a certain amount of inflammation is absolutely necessary, but the inflammation must be confined within certain limits." Subsequently, however, he modifies this assertion by admitting that, though inflammation be necessary for the formation of the bond of union, none is required for its organization, or its ultimate development into fibro-cellular tissue."

We object to this absolute proposition. We believe that even for the production of the reparative material, inflammation is by no means *necessary*, though it often does exist as a complication of the healing process. All that is requisite in this mode of repair, when the surfaces are not permitted to separate too widely, is a very thin layer or coating of lymph on each margin of the wound; and in all vascular and actively nourished tissues this may be provided without the least inflammation, as is often witnessed in our own persons, and still more commonly in the inferior animals. For each tissue possesses not only the power of maintaining its normal condition, but has beyond this a *reserve power*, as Mr. Paget expresses it, by which it is enabled to meet very many of the unusual exigencies and emergencies of life. Inflammation is often rendered necessary, even in parts abundantly supplied with blood, by wide separation of the margins of the wound, even the reserve power of the tissues being insufficient without inflammation to provide organizable material for the adequate filling up of the chasm, and it is still more essential in injuries of non-vascular structures; and, again, it is very frequently induced by undue irritation of the wound. But it must be borne in mind that the process of inflammation is otherwise very inimical to reparation; it occasions degeneration of structure in the margins of the wound, and renders the exudation for repair less susceptible of organization. So that it is only after the inflammation has ceased that reparation proper commences.

We are compelled to pass over a great deal which is interesting and valuable in Mr. Erichsen's teachings concerning *the varieties and complications of wounds.*

One of the striking peculiarities and merits of the book before us is, the amplitude of detail in which the author indulges in all practical matters; and in this respect it contrasts very favorably with all the other one-volume Expositions of Practical Surgery with which we have met. Mr. Erichsen's work is thus at once elevated above the rank of a mere text-book for students, into all the dignity and importance of a *vade-mecum* for the most experienced practitioner. We have a good illustration of this in the *tenth chapter*, in which an account is given of the phenomena, effects, and method of preventing and counteracting the dangers, of the accidental admission of air into a wounded vein. Our limits will not permit us to extract from this chapter, as we had intended to do. But we commend the whole to the attention of our readers. They will here find, in the compass of a few pages, a most capital summary of what relates to this alarming and frequently fatal accident; it has been condensed from the researches of French and English observers, as well as from an admirable paper published by Mr. Erichsen himself in "*The Edinburgh Medical and Surgical Journal.*"

The 14th, 15th, 16th and 17th chapters, are devoted to an elaborate and learned consideration of *Fractures*, *Dislocations*, *and other injuries of the Joints*, in all their varieties of simplicity and complication.

The following paragraph will commend itself to every sensible person: "Special apparatus should be employed as little as possible in the treatment of fractures. It is scarcely ever necessary in simple fracture, and is far more cumbersome and costly than the means above indicated, which are all that can be required. I have no hesitation in saying, that a surgeon of ordinary ingenuity and mechanical skill may be fully prepared to treat successfully every fracture to which he can be called, by having at hand a smooth deal plank, half-an-inch in thickness, and a sheet of gutta-percha, undressed sole-leather, or pasteboard, to cut into splints as required," with rollers of course, p. 190.

This we approve of most heartily; but we must confess that we cannot unite with him in his unqualified commendation of the *immovable apparatus*. After describing its various forms, and acknowledging his own former unwillingness to employ the starch-bandage in the early stages of fracture, Mr. Erichsen says: "During the last year, however, I have followed Seutin's plan in a great many cases, at least fifty or sixty, of fracture of all kinds, putting the limb up in the starch-bandage *immediately* on the occurrence of the injury, and have found the practice an extremely successful one, even in fractures of the thigh; so much so, that at the Hospital I now rarely use any other plan of treatment. I find that the moderate pressure of the bandages, aided probably by the great evaporation that goes on during the drying of so extensive and thick a mass of wet starch, and which produces distinct sensations of cold in the limb, takes down the extravasation most effectually, and enables the patient to leave his bed usually about the *third day* after the injury, when the fracture is in the leg or ankle, and about the sixth when the thigh is broken; so that very commonly we now treat all patients with simple fractures of the leg, and many of those of the thigh, especially in children, as out-patients." P. 191.

"The proof of the pudding is in the eating," as the adage says, and very properly, too; and as we have never seen a fracture of the thigh treated from the first after this method, we submit our doubts very diffidently, and with becoming deference to the decided approbation of Mr. Erichsen, M. Seutin, M. Velpeau, and other distinguished surgeons who advocate it. We say, therefore, that, however effectually the starch apparatus well applied may prevent or remove extravasation, inflammation and other such contingencies of fractures, these are really the least serious accidents which are to be apprehended. We have treated very many simple fractures, and we have rarely seen an amount of extravasation and inflammation which rest, aided or not by the application of cold or more stimulating lotions, has not removed in a few days. The inconveniences which are chiefly to be guarded against are, unquestionably, shortening and angular deformity; and these are to be strenuously combatted in all cases excepting in perfectly transverse fractures; and they will frequently require more than can be accomplished by the best contrived splints, aided by the most carefully maintained extension and counter-extension, the patient being the while prone upon his fracture-bed. We own that we shall feel unwilling ourself to trust to the starch apparatus, until we actually *see* the experiment successfully tried in the practice of another; we object to the patient being allowed to be up and moving about, to the limb being so perfectly concealed, and to the omission of extension and counter-extension.

Mr. Erichsen's remarks on the nature, causes, and treatment of "*Ununited Fracture*" are, we think, extremely judicious. The methods of treatment which he enumerates are the introduction of acupuncture needles, the subcutaneous section of the ligamentous connecting tissue by a tenotome, the introduction of a seton across the false joint, or gradually cutting through this by a silver wire passed around the point of fracture, and the employment of ivory pegs after the method of Dieffenbach. The operation of removing the false joint he very justly condemns. The methods which he considers as most advisable are the introduction of the seton, and of the ivory pegs. Concerning the first of these he remarks: "The introduction of a seton across the false joint, though occasionally successful, is apt to give rise to dangerous and even fatal consequences, from arterial hæmorrhage, erysipelas, diffuse inflammation and suppuration of the limb. The threads must not be left beyond a few days, when sufficient action will have been induced."

It appears to us that all these methods of treatment, excepting that of the subcutaneous section of the ligamentous medium of connection, and the employment of acupuncture needles, are open to the very serious objection that they virtually induce a condition approximating more or less closely to that of a compound fracture, the dangers of which we all know and fear. Dr. Brainard, of Chicago, has recently published in Paris a very interesting memoir on this subject, detailing a series of experiments and researches upon the effects produced by different foreign substances when retained in contact with bones. We cannot quote them here. He also describes a method of treating ununited fracture which, so far as we can judge, is more likely to be generally successful, and is infinitely more safe than the plans commonly resorted to. It consists in penetrating the integuments and the fragments of the bone close to the fracture with a sort of awl; the bone is pierced in various directions, and at several points, from one and the same opening in the integuments, the awl not being withdrawn until the different perforations of the bone are accomplished; then the orifice in the soft parts is carefully closed with collodion, and the limb kept at rest in the ordinary manner. (Mémoire sur le Traitement des Fractures non réunies, et des Déformités des Os, par Daniel Brainard, M. D. avec 19 figures, &c., 1854. P. 50, et seq.)

The almost uniform *eversion* of the foot in fractures of the thigh has always been a matter for comment. Mr. Erichsen has lately had an opportunity of investigating its cause in the case of an old man who died a few hours after having received a compound comminuted fracture of the middle and lower thirds of the right femur. "It was found that the gluteus maximus and medius could be divided without affecting the position of the bone; but when the *gluteus minimus* was cut across, it yielded somewhat. The *pyriformis* and external rotators were now felt to be excessively loose, and on cutting these across, the end of the fragment could at once be drawn inwards, all opposition ceasing."

In this country, certainly in this city, surgeons are accustomed to treat fractures of the thigh exclusively in the extended positions, and by means in most cases of Physick's and Hutchinson's modifications of Desault's apparatus. We have long thought, and, in our limited sphere, taught, that this exclusive mode of treatment is not the best. And we are glad to find our opinion confirmed by Mr. Erichsen. He recommends *four* methods: 1st. By simply relaxing the muscles of the limb, laying it upon its outer side, flexing both the thigh and the leg, and supporting both by an angular splint extending from the hip to the ankle, on the outer side, and by a short straight thigh splint on the inner side; 2d, by extension in the straight position, by means of Liston's splint; 3d, by the double inclined plane; 4th, by a starched bandage. The first method he adopts in those cases of "fractures about a couple of inches below the trochanters, in which there is a great tendency to the projection outwards of the lower end of the upper fragment." In these cases the deformity in question is undoubtedly due to the contraction of the gluteus maximus and gluteus minimus muscles, and we have never found any difficulty in successfully treating them with Physick's apparatus, by simply *throwing the whole of the injured limb out from the middle line of the body*, and thus making the line of the axis of the lower fragment correspond with that which the upper fragment had been compelled to assume by the action of the before-mentioned muscles. But we have seen fractures of the femur in which there was an angular deformity presenting anteriorly, due to the contraction of the psoas magnus and iliacus internus muscles which are inserted into the lesser trochanter. This deformity cannot be overcome by direct pressure upon the upper fragment, because it is difficult to act upon it with sufficient power to overcome the muscular force, and even if this could be done the fracture would probably be rendered compound from sloughing thus occasioned. We can, however, act upon the lower fragment by elevating it so that its axis shall correspond with the direction assumed by the upper. In such cases we would use the *inclined plane*, rather than any straight splint, and rather than place the limb in the flexed position, as in the first plan suggested by Mr. Erichsen.

Dr. Brinton has judiciously introduced a note here recommending Dr. Physick's apparatus, and also a recent modification of the same by Dr. T. Hewson Bache, of this city, which consists in the attachment of a screw to the transverse block of the long splint, so that extension may be accomplished gradually.

We shall offer no comments upon the chapter in which the other surgical injuries are considered, farther than to say that, so far as we have examined them, they are rich in the facts they contain, and in the evidence of Mr. Erichsen's intelligence and learning.

The *Third Division* of the book treats of *Surgical Diseases.*

It is related of a French Academician that on hearing Pinel's Classification of Diseases read, he exclaimed, "quel nombre prodigieux d'ennemis!" Our readers might well make the same exclamation, were we to enumerate the surgical affections which Mr. Erichsen very fully describes. But let them not be alarmed; we shall not put them to this "peine forte et dure."

The treatment of cancer is a matter of vast importance; and we pause in our necessarily hasty survey of Mr. Erichsen's elaborate discussion of the pathology and treatment of tumors, to see what his opinions are upon this subject. Concerning the treatment by *pressure*, which was tried by Mr. Young, in London, more than thirty years ago, subsequently by M. Recamier and M. Tanchou, in France, and very recently revived by Dr

Arnott, in England, Mr. Erichsen holds the following language: "The first question that necessarily arises in reference to the employment of pressure in these cases, is whether it can effect a cure. This it could only be expected to do by producing atrophy, and the subsequent absorption of the strictly local forms of cancer. The only case on record with any pretension to a conclusive character in this respect, is one related by Dr. Walshe, in his excellent 'Treatise on Cancer,' of the cure of a tumor of the breast, believed to be cancerous, by compression. But even this instance I cannot look upon as by any means conclusive; for although no one can entertain a higher opinion than I do of the very remarkable diagnostic tact of Dr. Walshe, yet I think there can be no doubt in the mind of any surgeon that it is absolutely impossible to determine in every case, by any amount of diagnostic skill, the true nature of a chronic tumor of the breast, and that, in fact, we constantly see the most experienced practitioners find after the removal of the tumor that it was of a different character from what they had previously anticipated. This difficulty attaches to Dr. Walshe's case, and I think that we possess no proof that the tumor of the breast which underwent absorption under the pressure of Dr. Arnott's apparatus, was of a truly cancerous nature, and that it might not have been a chronic mammary tumor or some similar growth, that we know will disappear under this kind of treatment." He continues his remarks by admitting that, if resorted to in the early stage, it may retard the progress of a cancerous tumor by diminishing the amount of blood which it receives, and by inducing absorption of inflammatory exudations. In other instances, he thinks that pressure does harm.

As regards the removal of a cancerous tumor by the knife, Mr. Erichsen says it should not be attempted when the cancerous cachexia is strongly marked, as evidenced by the existence of several tumors, by their rapid growth, &c.; and again, when the tumor progresses very slowly in old people, when the whole tumor, or the whole of the affected organ, cannot be removed, the operation, he thinks, should not be performed. "Those cases of cancer in which an operation is, in my opinion, not only perfectly justifiable, but should be urged upon the patient as affording the best prospect of preserving life, are, in the first place, those in which the disease has appeared to originate from a strictly local cause, in persons otherwise in good health, in whom there is no cachexia or hereditary taint. If the tumor be of a scirrhous character, slow in its progress, simple, distinctly circumscribed, without adhesion to, or implication of, the skin or glands, and more especially if it be attended with much pain, or with immediate risk to life from any cause, and if the whole of the growth, together with a sufficient quantity of the neighboring healthy tissues can be removed, most surgeons of authority in these matters would consider it to be a fit case for operation."

Mr. Erichsen does not allude to the treatment by ligature of the vessel or vessels supplying the tumor, when the latter is favorably situated for the operation, as in the case of the parotid gland for example.

In his chapter on *Syphilis*, Mr. Erichsen is rather too lavish of *assertion* without attempting to sustain his statements by his own observations, or those of other careful men. Thus, he speaks of inoculation as being a "*sure test of primary syphilis*," and again as a "*sure and unerring test.*" This it certainly is not; cancer cannot *always* be inoculated, just as the matter of small pox or of vaccinia cannot always be inoculated in persons unprotected. The experiments of many investigators prove this.

Again he says, "constitutional syphilis is not contagious. This point which I look upon as one of the fundamental doctrines of syphilis, has, in my opinion, been incontestably proved by the observations of Hunter, and more recently by Ricord, who has shown that the pus from secondary sores is never inoculable; observations that are fully carried out by what may often be observed in practice."

The experiments of Mr. Hunter and M. Ricord are very good as *negative facts;* but since they were performed, other experimenters have apparently arrived at *positive results in favor of the contagiousness of constitutional symptoms;* if we can rely on the statements of M. Weller, of Prague, and M. Vidal, of Paris, the actual contagiousness is proved. And Mr. Whitehead, in his interesting book on Hereditary Diseases, narrates a case in which constitutional syphilis was developed in a perfectly healthy child of perfectly healthy parents, by vaccination with matter taken from the arm of another child who had the disease, unknown, at the time, to the physician and the other parties. Even admitting that a few such cases are not *conclusive*, they still render it best to speak less positively against the contagiousness of constitutional syphilis, than Mr. Erichsen has done.

In the chapter on *Diseases of the Veins*, the author recommends the following method of treating *varix:* he passes hare-lip pins at several points underneath the vein, places a piece of wax-bougie upon the vessel, and then twists a strong thread in the form of the figure *eight*, (8) around all; the vein gradually becomes obliterated.

Nearly one hundred pages are devoted to the study of *Aneurism.* The reader will here find a very learned and admirable exposition of the whole subject in its scientific aspects, together with ample and instructive details concerning the pathology and treatment of special aneurisms. The history of the various modes of management of this important disease is narrated with considerable minuteness, and with more than usual accuracy.

There can scarcely be, at this day, any doubt as to the value of the treatment of aneurism by compression ; yet we confess that we turned with some curiosity to the section in which Mr. Erichsen deals with this matter, knowing that the surgeons of the British Metropolis are not always ready to adopt the practice, however judicious it may be, of their provincial brethren. But upon this point Mr. Erichsen is as judicious and as decided as we expected to find him. He says, "After carefully considering the relative merits of the two plans of treatment, I think we may conclude that, though in some few instances neither method can be adopted, and amputation is the sole resource, yet, that in others, compression can be employed when it would not be safe to have recourse to the use of the ligature ; and that in all ordinary cases of femoral and popliteal aneurism especially, compression should be preferred to the ligature, inasmuch as it is not a more tedious, and an infinitely safer method of cure. At the same time it must not be forgotten, that its success depends very greatly upon the continuous care bestowed on the case during the progress of the treatment."

Were we asked to point out the most meritorious part of Mr. Erichsen's book, we should select this. Indeed we know not where to find a better disquisition on aneurism, general and special, than is here presented.

The signification of "*Necrosis*" is, we think, very justly expressed in the following sentence: "The transition from caries to necrosis is easy; *caries* may be regarded as the granular disintegration or molecular death of the osseous tissue, conjoined with suppuration of the surrounding parts; whilst *necrosis* must be looked upon as the death of the osseous tissue as a whole, a condition indeed closely resembling that of gangrene of the soft parts." But in setting forth the *mode of reparation* after necrosis, Mr. Erichsen is, it appears to us, very much at fault. He says, "When the outer lamellæ alone are necrosed, new bone is deposited by the surrounding periosteum, and the depression that has formed on the surface of the old bone is filled up by a kind of cicatricial fibrous tissue, which ultimately ossifies. If the whole of the inner lamellæ of the shaft die, constituting central necrosis, the outer layers of bone become greatly consolidated and thickened by osseous matter deposited from the periosteum, in which, in the majority of cases, the circular or oval apertures termed *cloacæ* form for the ultimate extrusion of the sequestrum. When the whole of a shaft dies, the reproduction takes place from various sources, principally perhaps from the periosteum, and the medullary membrane, if that is left, which become inflamed, vascular and detached from the necrosed bone; the periosteum indeed takes the principal share in the reproduction, and has been considered by some pathologists as the true organ of reproduction of new bone. Then again the soft tissues of the limb generally, if thick, as in the thigh, contribute to the formation of plastic matter, which generally ossifies, and so tends to strengthen the new case; and, lastly, the articular ends of the old bone still preserving their vitality, throw out sufficient osseous matter to consolidate themselves firmly to the new shaft that is formed.—Thus it will be seen that the new bone is formed by the vascular and healthy tissues generally that surround the seat of disease, though in this reparative action the periosteum and medullary membrane take the chief share."

Mr. Erichsen ignores entirely any part which the bone itself takes in the reproduction; yet he states in another place that the loosened sequestrum is extruded by the growth of granulations which sprout up from the subjacent bony surface. Now, if we do not greatly err, it is to the ossification of these very granulations that reproduction of bone is largely due.

The idea that the periosteum is chiefly concerned in the repair of fractures and necrosis probably originated in the observation, that the bloodvessels before penetrating to the bones ramify in the periosteum, and that if this membrane be removed to any extent the superficial lamellæ of the bones die. But the chief function of the periosteum seems to be to provide a bed in which the nutritious vessels destined for the bones may be broken up and uniformly diffused before actually entering the osseous structure; consequently when this membrane is removed, the nutritious canals are separated with it from the bone, and the latter suffers from the cutting off of its supply of nutriment.

We are aware that many observers and writers of authority maintain not only that the periosteum is the chief, but that it is the only source of reproduction and repair. And the experiments made by Syme and Mr. Stanley have recently seemed to throw great weight into this scale. But we think a careful perusal of the observations of Miescher, Müller, Goodsir, Lebert, and many others, will show that, as might be expected from analogy with what we know to be the case in other organs, to the bone itself is committed the chief care of maintaining its normal structure, and repairing its own injuries and diseases.

Several well written pages are occupied with an account of *Diseases of the Jaw.* Mr. Erichsen has fallen into the very common error of believing "that the operation of *excision of a portion of the lower jaw* for tumor of that bone was first performed by Dupuytren." Dupuytren's operation was accomplished in 1812; but Dr. Deadrick, of Tennessee, performed a similar feat in 1810, and the account of this was published in the *American Medical Recorder*, vol. 6. Mr. South, in his edition of Chelius' *System of Surgery*, vol. 3, p. 745, admits the priority of the American operation, and a still more circumstantial vindication of the same was published by Dr. Blackman, in the New York Journal of Medicine, 1852.

Mr. Erichsen discusses very fully indeed the pathology and treatment of *Hernia* and the allied obstructions of the alimentary canal, treating not merely of the accident as it most commonly appears, but elucidating its irregularities and its complications, and explaining the various methods of combatting them.

Concerning the *operation without opening the sac*, he thus expresses himself: "The possibility of removing the stricture in strangulated hernia without laying the sac open, naturally suggested itself, when it was known that in many cases the stricture was seated in the tendinous and cellular tissues outside the neck of the sac, and that when these were divided the protrusion was readily reduced. This operation was performed by Petit as long ago as 1718, but was seldom practised until it was revived of late years by Aston Key and Luke. The great advantage sought to be gained by this operation is, that as the peritoneum is not interfered with or its cavity opened, the risk of peritonitis will be proportionally lessened. The wound made by the operation being altogether superficial, and the sac not opened, its risk has been compared to that of the taxis, with the addition of what would result from a superficial wound. This argument would be conclusive in favor of the operation without opening the sac, could it be shown that in all cases of strangulated hernia the peritonitis is occasioned by interfering with the peritoneal cavity; it must, however, be admitted, even by the advocates of Petit's operation, that this is not the case. In many instances the inflammation exists before the operation is performed, being evidently produced by the stricture of and injury to the gut. But it cannot with fairness be argued that, though the peritonitis may exist before the operation, the incision of the peritoneal cavity does not increase it. Even in healthy persons, laying open the abdomen, handling the gut and the omentum, and pushing the fingers into the peritoneal sac, would always be followed by intense, perhaps even fatal, peritonitis. It is only reasonable to believe that the same procedures in an already inflamed peritoneum would be followed by equally disastrous results."

He then proceeds to examine the *pros* and *cons* more in detail, presenting the statistical results of the examinations into the question as conducted by Mr. Luke, and concludes as follows: "For the various reasons that have been mentioned, I am decidedly of opinion that this operation should always be attempted in preference to the ordinary one of opening the sac, in those cases in which the hernia has not been long strangulated, presents no sign of the occurrence of gangrene in it, and more especially when it is femoral. Even if the surgeon fail in completing Petit's operation, in consequence of the incarceration of the stricture in the neck of the sac, or the constriction of this part, no harm can have resulted; for the sac, after being exposed, may at any time be opened in the ordinary way, and the operation be completed by dividing the stricture from within."

This mode of operating has within a few years past had some very earnest and able supporters in Messrs. Bransby Cooper, Key, Luke, and Gay, who urge its advantages very forcibly and support it by seemingly strong statistical authority. But it must be remembered that it is in precisely such affections as strangulated hernia, that statistics must be sifted with the closest scrutiny and suspicion, because it is so excessively difficult to array against each other cases which are sufficiently analogous to admit of a fair comparison, as respects the age, constitution, and health of the patients; the duration and intensity of the strangulation; the size and other peculiarities of the hernial tumor; the amount, continuance, and nature of the efforts to secure reduction previous to the operation, &c. &c. And, moreover, in contrasting cases operated upon by the same surgeon, by Mr. Luke, for example, in some of which the sac was opened and in others unopened, it is important to remember this fact, viz., that the sac was opened because the operator deemed it necessary to do so in consequence either of being otherwise unable to reduce the gut, or of finding such appearances of the sac as induced him to fear the existence of greater organic changes of the bowel itself—either of which circumstances would have increased the fatality if Petit's operation alone had been performed. This proceeding, therefore, is confessedly not applicable to all cases; and we think that the difficulty if not the impossibility of certainly determining, without opening the sac, whether the bowel is in a fit state to be returned, or whether there may not be bands of adventitious tissue or other obstacles to the restoration, constitute serious objections to the operation. It is a question, too, whether the opening of the sac really is such a fruitful cause of peritonitis as is generally supposed; for the amount of mortality is not directly proportioned to the extent of peritoneum and of intestine exposed—

the smaller hernia being those which are most fatal. The intensity and extent of the peritonitis result rather from the constriction for which the operation is resorted to, as Mr. Erichsen observes in the passage cited. Mr. Key himself says, "In tracing the inflammation consequent upon the operation for hernia, it is found to spread from that portion of the bowel that has been strangulated, over the peritoneal surface of the intestines, and not to have its origin from the incision of the sac, although two wounds are usually inflicted upon it. The peritoneum, about the seat of stricture exhibits fewer signs of acute inflammation than the investment of the bowels," &c., although this portion of the peritoneum next to the sac is, of course, most exposed to the influence of the air when opened. Again, inflammation of the peritoneum is by no means the only, or perhaps not the most frequent cause of death; "of 52 fatal cases cited by Mr. Gay, the symptoms of peritonitis alone existed only in eight, and in these the symptoms were so slight as to lead to the supposition that the patients died from the shock to their systems, rather than from the peritonitis."

We offer these few strictures upon Petit's operation as a caution against admitting too implicitly and hastily the authoritative recommendation of Mr. Erichsen. The reader will find the question very ably and fully discussed by Mr. Hancock, in a little book published on the subject in London, in 1850.

From the author's full and satisfactory description of the *Diseases of the Genito-urinary Organs,* we shall cite what he says concerning "the *Perineal section*" in case of stricture, as recommended by Mr. Syme,—a proceeding which has been, so far as we can hazard an opinion, very unnecessarily and injudiciously opposed. After detailing the mode of performing this operation, the cautions to be used, &c., Mr. Erichsen says: "I look upon this operation of urethrotomy as a very decided advance in the treatment of certain forms of stricture. It is easy of performance and successful in its immediate results. In Mr. Syme's hands scarcely any accidents have occurred out of 77 cases in which, up to the present time, he states that he has performed it. As my experience is very limited in comparison to his,—as up to the present time (May, 1853) I have only had occasion to do it five times—I can speak with less confidence; but so far as my observation goes, I have met with no difficulty or danger in the division of the stricture. Although fatal cases have occasionally been recorded, yet this is nothing more than we must expect to happen from time to time in any operation that is performed upon the urinary organs whilst diseased, more particularly if there be a granular condition of the kidneys,—a state of things in which I have known the simple introduction of the catheter to be followed by death in six or seven hours," &c. He then states the classes of cases in which "urethrotomy may be advantageously employed," and the conditions which render it proper. With reference to the permeability of strictures,—a point of essential importance in the performance of Mr. Syme's operation, Mr. Erichsen says, "impermeable strictures, though frequently spoken of, are, however, I believe, very rarely met with. Mr. Syme, indeed, denies their existence, and states with much truth, that if urine can escape through a stricture, a bougie can be got in. I must admit that I have never seen a truly impermeable stricture, either during life or after death." p. 821.

We had marked passages in several other chapters which still remain, upon which we had thought of commenting, and others which we desired to quote. But our limits are, we fear, more than reached.

In conclusion, we must do Mr. Erichsen the justice to say that, although his book is not free from faults, particularly in matters relating to the Principles of Surgery, it is, in our humble opinion, the best treatise on "the Science and the Art of Surgery," in any thing like the same compass, which we have seen. His style is very good, clear, precise, and not verbose. The illustrations are excellent and very well executed, and the whole mechanical part of this particular edition is better than that of most of our reprints.

Dr. Brinton has added a few notes, relating chiefly to Operative Surgery, in which he has manifested much judgment. We observe that in them he refers generally to the practice of his distinguished preceptors, Drs. Mütter and Pancoast, whose opinions, of course, are entitled to great consideration.

*Epilepsy and other affections of the Nervous System, which are marked by Tremor, Convulsion or Spasm; their Pathology and Treatment.* By CHAS. BLAND RADCLIFFE, M. D., &c. London, 1854.

The author, to whose politeness we are indebted for a copy of his work, prefaces his remarks upon epilepsy and the cognate disorders by some preliminary considerations upon the physiology of muscular contraction, as it is manifested in its three principal forms—in ordinary muscle, in the coats of vessels and in the heart. In each of these instances contraction is generally supposed to be called into exercise by certain stimuli, vital or physical. Many doubts, he says, as to the correctness of this view "are self-evident, and among these the more obvious are those which are suggested by *rigor mortis*, and by that contraction which occurs in the muscle-like tunic of the dartos under the influence of cold." *His* view is "*that all stimulants, vital and physical, antagonise muscular contraction, and that contraction happens from ordinary molecular attraction, when the muscle is not irritated.*"

As regards the induction of muscular contraction by different stimuli, as manifested in ordinary muscle, it is argued that the stimulus of nervous influence is unnecessary to contraction, because the involuntary muscles are far more sparingly supplied with nerves than the voluntary, and yet their power of contraction, as measured by its duration, is indefinitely greater. In reptiles also, in which the nervous centres are sparingly developed and the blood is scantier, muscular contraction is found to be a more marked phenomenon than in animals of higher rank. As regards the effect of simple contact as one of the stimuli, he says, "that it is difficult to acquiesce in the common belief that the hollow involuntary muscles are *excited* to contract by their natural contents." In the stomach and alimentary canal the food remains quietly until digestion is complete. Contraction begins when those molecular changes are at an end which could have acted as a stimulus to the muscle. So, in regard to the uterus and fœtus. The latter, instead of exciting the uterus to contract, grows and causes the uterus to expand by the stimulus of its living presence, until the growth begins to trench upon the supplies necessary for the proper nourishment of the mother. "Then the child becomes a source of exhaustion to the parent, and this exhaustion reacting upon the uterus, brings back the state of contraction; for if the uterus went on expanding in consequence of stimulation, it must needs return from that state of expansion if the degree of stimulation be diminished, and this equally, whether the diminution be occasioned by the death of the child, or whether it be the result of the child having lived until it begins to starve the mother by its too clamorous wants." Nor is muscle, he says, *excited* or irritated to contract by the contact of a needle or any other body. The analogies existing, not only between the contraction of a muscle and the discharge of the electrical apparatus of the torpedo, but also between the organic structure of the muscle and that of the apparatus, render this very improbable. "These analogies lead to the *impression* that the needle does not excite or irritate the muscle to contract, but that it procures an electrical discharge similar to that which takes place from the battery of the fish under similar circumstances, and the impression receives strength from the fact (which has been fully demonstrated by M. Dubois Reymond) that electricity is present in the muscle *before* contraction, which departs during contraction." The needle merely furnishes a channel for its escape.

The primary fact, in regard to the influence of electricity upon muscular action, is stated to be, "that a living muscle deflects the needle of the galvanometer, produces electrolysis, and affords other evidences of sensible electricity, while it is *at rest*, and that it ceases to evince any sign of such action *during contraction*. This fact—the latter part of which has been recently discovered by M. Dubois Reymond—is of primary and supreme importance, and a full and definite conception must be obtained respecting it, before proceeding to investigate the influence of artificial electricity upon muscular action." Several pages are devoted to the experiments of MM. Reymond and Matteuci on muscular contraction, as influenced by electricity, the former proving "that the contraction is accompanied by the disappearance of the innate electricity of the muscle and nerve, and the latter showing that artificial electricity induces contraction by diminishing or neutralizing and not by intensifying this innate electricity." The author contends also that muscular fibre cannot be stimulated to contract by both heat and cold, as is generally said to be the case. "Cold cramps and stiffens the hands; warmth relaxes them. Nor is it correct to say that heat is produced during muscular contraction. It is produced during muscular *action*, as during the act of sawing; but this action consists of alternate relaxations and contractions. It is possible, therefore, that the heat may be due to the relaxations, nay it is probable, for when the limb is wearied and the action over, and when the temperature is highest, the muscles are relaxed. The fact also that the heat originating under these circumstances continues for a long time after the exertion has ceased, is an argument that it is due to some other cause than muscular contraction." The stimulus of the light upon the iris is explained by the adoption of Bichât's view, that the closure of the pupil is due "to the expansion of the entire curtain of the iris, and not by the contraction of its inner rim." So also is it denied that chemical agents can act as stimuli. The first chapter concludes as follows: "Contraction, then, as seen in ordinary muscles would appear to be analogous to that contraction which takes place in inorganic bodies on the abstraction of heat, with this only difference that more forces have to be abstracted from the organic than the inorganic body. The analogy is indeed perfect; for even that remarkable degree of contraction which is witnessed in muscle, as compared with that which is seen in inorganic bodies, may be a natural consequence of the physical constitution of muscle; for as muscle is composed almost exclusively of certain gaseous elements, it *may* contract to a great degree under a small abstraction of heat, because it is the law of its constituent gases, as gases, so to contract."

The second chapter is upon muscular contraction as manifested in the coats of vessels. The author argues that the condition of the capillaries is opposed to the idea that vital stimuli produce contraction. Joy flushes and fear blanches the skin. In plethora the vessels are red and full; when the blood is poor and watery, as in anæmia, they are shrunk and empty, all the vessels dilating, in fine, in proportion to the amount of vital force expended upon them. Physical stimuli are similar in their effects, as instanced by the hand becoming full and flushed when held to the fire. On these grounds he presumes the existence of a capillary force, independent of the heart, but co-operating with it in facilitating the progress of the blood. The vessels, he thinks, are not composed of ordinary solids, but are, in reality, gases coerced into solidity, and are therefore more affected by heat than the fluids within them, whose chief constituent is water. The different ratio of expansion in the two, leaves *vacua* between them, so constraining, as it were, the blood to enter into them. Inflammation is due to over expansion of the vessels by heat or other agents.

The third chapter takes up the subject of muscular contraction as manifested in the heart. That there is an active power of dilatation in this organ is shown by the absence of all elastic tissue, by the resilience of which the heart might recover itself after the systole. As a proof of this statement the state of the heart in insects is referred to. In them, the heart, lying in the general cavity of the body, floats in the sanguineous fluid which fills the cavity; it has no venous trunks, the blood entering through simple valved slits in the walls; in addition it is a lax, membranous tube. Hence, an active power of dilatation must exist to overcome the pressure of the surrounding fluid. Again, under the influence of fear the heart beats hastily, yet little blood is propelled by it, the skin being cold and pale. It is thought probable, therefore, that the chambers of the heart are diminished in size by the contraction of the walls, when nervous power is depressed. The effect of blood upon the action of the heart, it is stated, is irreconcilable with the ordinary views held by physiologists. That the systole is not excited by it is shown by the fact that the heart remains distended with blood during a full half of the time occupied in its rythm. In plethora, where the pulse is full and slow, the systole is deferred; in anæmia, where it is empty and quick, the blood is expelled immediately. The arterial stream, he argues, setting out at the systole, developes the nervous influence at its proper sources, and passing along the nerves to the heart, induces the diastole and thus causes the systole, by cutting off the supply of blood, suspending, for a time, the active development of nervous influence. The effects of physical agents are similar to those of vital agents. Heat stimulates, the opposite of contraction, and cold diminishes the size and suspends the beats of the heart of the fœtal chick, and heat restores and renews them.

If muscle contracts, the author says, when stimulated, the phenomenon is peculiar to living bodies, and is unintelligible on physical principles. "If, on the other hand, it contracts because it is *not* stimulated, or, in other words, because some stimulus has been removed, which had prevented the natural molecular attraction of the tissue from coming into play, then the phenomenon becomes analogous to the contraction which takes place in a bar of metal, or in any other inorganic body, on the abstraction of heat."

The occurrence of rigor mortis, the muscular rigidity which sets in after death, is thus explained, as being the result of molecular attraction : It cannot be referred to some lingering vitality in the muscles, for the time of its accession and the degree of this vitality are inversely related to each other ; it takes placc soonest in old age and where disease has been chronic, as in consumption, and occurs later and longer where the person has been cut down in the full vigor of health. M. Brown Séquard has shown, by numerous experiments, too, that by the injection of warm blood the muscle may be made to relax and recover its lost irritability.*

The second part of the work is devoted to the consideration of Epilepsy and other affections of the nervous system, marked by tremor, spasm or convulsions. The skin in epileptics is pale and sallow, or dark and congested, the pulse feeble, the circulation always deficient in power. After a convulsion reaction sets in with great rapidity, but this is always *after the convulsion.* The intellectual and memorial faculties are deficient, and insanity is very common. The slowness with which the system rallies after fatigue and the pallid and soft condition of the muscles also present unequivocal evidences of inactivity. These considerations seem to prove, according to the author, that the convulsion depends "upon *want* of vital stimulation, which want had allowed the molecular attraction of the muscles to come into play and gain the ascendancy."

In the cognate affections, chorea, delirium tremens, subsultus of fevers, mercurial poison, hydrophobia, convulsions of fever, lead and prussic acid poisoning, uremia, cholera cramps, tetanus, ergotism and catalepsy, the circulation is also wanting in power,

* In this the author is mistaken ; *warm* blood is *not* essential ; the heat used was 66° Fahr., the temperature of the atmosphere.

and approaching syncope or asphyxia. The condition of the nervous system, too, as reflected in the intellect and senses are, as a necessary collorary of a weak circulation, feeble and defective. The entire history of these affections is, in fine, "at complete variance with the idea that the muscles are provoked to excessive contraction by excessive stimulation. It is as much at variance with the hypothesis as it is in harmony with that doctrine of muscular contraction which was propounded at the commencement of this enquiry, which doctrine is, that all stimulants, vital and physical, antagonise muscular contraction, and that contraction is brought about by ordinary molecular attraction when the muscle is *not* stimulated."

The phenomena of periodicity, diurnal, lunar and annual, as manifested in the lives of plants and animals, are also brought forward as proofs of the same law. The lower the grade of organization the greater is the disposition to periodicity. Plants are more obedient to the law than animals, being more deficient in that inherent life by which the animal resists the influences of season. Consequently, when signs of morbid periodicity are manifested in man, they must be regarded as evidences of a low condition of vital power, "a simple want of innate strength." These morbid signs exist in all convulsive affections.

The proper treatment of epilepsy and its allied affections deduces itself from the above considerations. All causes of depression and exhaustion must be avoided; nourishing diet and, above all, *stimulants*, in correspondence with the physiological and pathological views entertained of the disease, are strongly advocated, and we are informed that the writer has had every reason to be satisfied with the results. He has never met with a patient (epileptic) who has not been benefitted by such a course, and many have been completely cured.

Our limited space has obliged us to omit many illustrations and arguments adduced by the author. The remarks we shall make upon the subject must, from the same reason, be brief. His theory may be stated thus: Two forces exist in the production of muscular action. One of these agents is the action of stimuli, comprehending under this term the vital powers of innervation, of the blood, &c., and all physical and chemical stimuli. The other is molecular attraction, similar in its nature to the same force as exhibited in all inorganic substances, and evidenced by the post-mortem condition of rigor mortis. During life these forces antagonise, and are in direct opposition to each other. The application of this theory is, that convulsions, spasms and tremors are due to the action of molecular attraction, which has obtained the ascendancy in consequence of the absence of a want of due power of the proper stimulus.

In opposition to this theory, and as proof that contraction is an *active* power, the result of applied stimuli and not the effect of exhaustion, we shall cite a few examples. Urinary calculus occasions an almost constant desire to urinate. The contractions, it being immaterial to the argument whether these are produced by simple contact or through reflex action, are so frequent and powerful as to greatly diminish the cavity of the bladder and finally to produce hypertrophy of its walls.

In post-partem hæmorrhage, due to atony of the uterus, occasioned by the irritability of the organ being exhausted by powerful labor pains or other causes, contraction of its walls, it is well known, is brought on by passing the hand into the cavity and there irritating the internal surface.

In regard to the action of the heart, the author's argument that it is not excited by the presence of blood in its cavities we are willing to admit. That it is in any way *dependent* on nervous influence, we as unequivocally deny, for its movements are seen to take place in embryos before a nervous system has appeared, and while its walls are still composed of cells, and in cold-blooded animals long after its removal from the body has been accomplished.

The true cause of the hearts' action, we believe is to be found in the blood distributed to the *substance* of the heart and not its interior, as maintained by Haller, and we have adopted the explanation of Dr. Brown Séquard that the principal cause of that action is carbonic acid in the blood, as everything which increases the formation of that agent, increases also the frequency of the hearts' action, as seen in asphyxia, injection of carbonic acid into the blood and the phenomena produced by artificially holding the breath. The insufflation of oxygen, on the contrary, diminishes the frequency, but at the same time prolongs their duration. That carbonic acid is capable of acting as an excitant to muscular fibres is seen in the tremors, if not convulsions, that always occur when that agent is brought to bear upon them, as witnessed in the expulsion of the contents of the bowels and bladder after death, the extrusion of the fœtus in utero under similar circumstances and the spasmodic action in epilepsy, where black blood is present and in those recently dead from cholera asphyxia, to which may be added also, the violent peristaltic action that may be produced at will, by alternately arresting and permitting respiration to take place.

The effects of excitants is seen also in the contractions of the bladder when highly acid urine is present, and in the tenesmus of dysentery, occasioned by the action of irritating secretions upon an inflamed tissue.

The author states that uterine contractions take place at term in consequence of the exhaustion of the mother through the growth of the child. This assumption is disproved by the fact that women, in the last stages of phthisis, will often carry their children to full term and even then have prolonged labours, in consequence of want of contractile power in the womb—a fact which ought not to be, if contractility depended upon exhaustion.

The exciting cause of labour is still, however, an unsettled point. It is most probably due to some change in the placenta, by which its functions, as a respiratory organ to the fœtus, are affected. This change in the placenta affects its attachment to the uterus, and then it acts like any other foreign body,—a blighted ovum for instance—as a stimulus to the womb. This view is sustained by the analogies presented in the lower order of animals; by the detachment of the leaf, the fall of ripe fruit, &c.

Nor have we any reason to suppose that molecular attraction is the cause of rigor mortis. M. Brown Séquard's experiments have shown that this condition made its appearance very early in rabbits, and endured but a short time, when they were submitted to a powerful electro-magnetic current. When feebly electrized, it came on much later.—The difference in time was between 25 minutes and 144 hours.—The remarkable contractions, frequently witnessed after death from yellow-fever and cholera, to which the attention of the profession has been mainly directed by the researches of Dr. Bennett Dowler, of New Orleans, may be also adduced here.—In all his cases contraction followed by a gradual and uniform relaxation, ensued, when the muscles and more particularly the flexors, were struck by the hand or other substance. If several blows rapidly followed each other, the contractile function was soon exhausted. If the part were struck very forcibly, the contractility was killed almost immediately. These facts and M. Séquard's experiments prove incontestibly, we think, the existence of an inherent irritability of muscle—molecular attraction would certainly not be interfered with by such means.

In reading the book, we had marked several passages requiring comment—we can but allude to two of these—one is the statement, when speaking of muscular contraction, as manifested in the coats of vessels, "that the solids (of the body) acted upon (by heat), remember and obey the law of expansion which belongs to their constituent gases as gases." We need hardly remark that this statement is incorrect. All gases expand in the same ratio by equal increments of heat, and there is no proportion whatever, between the rates of increase of a substance in a gaseous and of that in a solid state.—Of course, a fortiori, there can be none between the said substance in its gaseous state and in that in which as a solid it is in combination with many other elements. In fact, there is no connection between the physical properties of an organic substance or other compound and its constituents.

Again, he thinks it difficult to believe that a muscle is irritated to contract by the contact of the point of a needle," and at the same time to remember the analogies which are known to exist, not only between the contraction of a muscle and the discharge of the electrical apparatus of the torpedo, but also between the organic structure of the muscle and that of the apparatus. These analogies lead to the *impression* that the needle does not excite or irritate the muscle to contract, but that it procures an electrical discharge similar to that which takes place from the battery of the fish under similar circumstances." We are altogether unable to see these analogies. Dr. John Davy, a reliable authority, in his account of "Some Experiments and Observations on the Torpedo," remarks (page 24) that his experiments have led him to the conclusion "that it is requisite to touch the opposite surfaces of the electrical organs or organ, or a conductor or conductors connected with them to receive a shock." This statement we regard as very important, as it clearly disproves any similitude between the two actions. Again, where is the analogy between the organic structure of a muscle and that of the electrical organs of the torpedo? In page 33 of the same work, the author remarks, "Reflecting upon the facts and observations which I have just detailed, it appears to me very difficult to resist the conclusion, that the electrical organs of the torpedo are not muscular, but columns, formed of tendonous or nervous fibres, distended by thin gelatinous fluid."

We had intended to have made a few remarks upon the author's views on Periodicity and on the treatment of convulsive affections. We fear, however, that we have already too much trespassed upon the good nature of our readers, and shall conclude by stating our opinion, that these different affections mentioned are due, not to the disturbing or counter-effects of vital power and molecular attraction, but either to an inherent faulty innervation, or to a deteriorated state of the blood. Either of these conditions is rarely found to exist alone. They react upon and produce each other, either directly or indirectly. Faulty innervation interferes with healthy nutrition, and the blood is, as a consequence, rendered unfit for the office it has to perform. If, on the other hand, the nervous centres are supplied with deficient or diseased blood, their functions must be performed in a feeble or irregular manner.

Disagreement is unpleasant at all times; more particularly when it is directed against opinions which appear to have been founded on an honest enquiry after truth. The author has, however, dealt so largely in assumptions and special pleading, that we have felt it a duty to speak plainly. In favor of his book, we may say that it is ingenious and well-reasoned. The subject, too, is one that is by no means settled, and erroneous as we believe his theory to be, we can heartily commend it as the product of a thoughtful and original mind.

---

*Woman: Her Diseases and Remedies. A series of letters to his class.* By CHAS. D. MEIGS, M.D., Professor of Midwifery, &c. 3d edition, revised and enlarged. Blanchard & Lea, 1854.

It is not our intention to enter upon any analysis of the work before us. We shall merely say that, it is distinguished alike by its sound judgment and intimate acquaintance with the subjects of which it treats. No one who reads it, can doubt that its author is an accomplished and erudite physician. As regards its style, to which the author modestly and deprecatingly alludes in his preface, we cannot agree with him in thinking any apology necessary. We deem it not less marked by its originality than by its sparkling, graceful and gentlemanly tone. There is, it is true, an occasional florid extravagance of language, to which much exception has been taken. This is a matter of too slight importance, however, to justify the severe cavils directed against it. Such disparaging and trifling criticism is an unworthy return for a work of so much value, and we are surprised and regret to see so much stress laid upon it.

That the work has been highly appreciated by the profession is evident from the fact, that the present is the 3d edition. We sincerely congratulate the author upon this success. It is his fair due. He has labored manfully and nobly in his vocation and in the midst of an extensive and distracting practice, has found time, through his determined industry alone, to give us this result of his matured experience. That he may live many years to instruct and delight us with his ready and pleasant pen, is the sincere and earnest wish of his numerous friends. The present edition has been much enlarged and carefully emended.

---

*A Universal Formulary: Containing the methods of preparing and administering officinal and other medicines. The whole adapted to Physicians and Pharmaceutists.* By R. Eglesfeld Griffith, M. D. A new edition, carefully revised and much extended by Robt. P. Thomas, M.D. Blanchard & Lea.

We are pleased to see another edition of this well and favorably known work. There are few physicians who will not find it a useful and valuable auxiliary. About 70 pages of new matter have been added. The high and deserved reputation of the editor for accuracy and research, are a sufficient guarantee that the addenda are entitled to the confidence of the profession.

